# *ASGR1* and *ASGR2*, the Genes that Encode the Asialoglycoprotein Receptor (Ashwell Receptor), Are Expressed in Peripheral Blood Monocytes and Show Interindividual Differences in Transcript Profile

**DOI:** 10.1155/2012/283974

**Published:** 2012-08-02

**Authors:** Rebecca Louise Harris, Carmen Wilma van den Berg, Derrick John Bowen

**Affiliations:** Institute of Molecular and Experimental Medicine, Cardiff University School of Medicine, Heath Park, Cardiff CF14 4XN, UK

## Abstract

*Background*. The asialoglycoprotein receptor (ASGPR) is a hepatic receptor that mediates removal of potentially hazardous glycoconjugates from blood in health and disease. The receptor comprises two proteins, asialoglycoprotein receptor 1 and 2 (ASGR1 and ASGR2), encoded by the genes *ASGR1* and *ASGR2. Design and Methods*. Using reverse transcription amplification (RT-PCR), expression of *ASGR1* and *ASGR2* was investigated in human peripheral blood monocytes. *Results*. Monocytes were found to express *ASGR1* and *ASGR2* transcripts. Correctly spliced transcript variants encoding different isoforms of ASGR1 and ASGR2 were present in monocytes. The profile of transcript variants from both *ASGR1* and *ASGR2* differed among individuals. Transcript expression levels were compared with the hepatocyte cell line HepG2 which produces high levels of ASGPR. Monocyte transcripts were 4 to 6 orders of magnitude less than in HepG2 but nonetheless readily detectable using standard RT-PCR. The monocyte cell line THP1 gave similar results to monocytes harvested from peripheral blood, indicating it may provide a suitable model system for studying ASGPR function in this cell type. *Conclusions*. Monocytes transcribe and correctly process transcripts encoding the constituent proteins of the ASGPR. Monocytes may therefore represent a mobile pool of the receptor, capable of reaching sites remote from the liver.

## 1. Introduction

The asialoglycoprotein receptor (ASGPR) (also known as the Ashwell receptor) mediates the capture and endocytosis of galactose- (Gal) and N-acetylgalactosamine- (GalNAc) terminating glycoproteins. The relevance of this function has been the subject of much debate; the primary role may be the removal of potentially hazardous glycoconjugates arising from normal tissue turnover, tissue injury, disease, and other causes [1]. Studies using knock-out mice have provided evidence for direct involvement of the ASGPR in removal of abnormally sialylated plasma glycoproteins. Mice lacking the sialyltransferase ST3Gal4 showed prolonged bleeding which was attributed to ASGPR-mediated clearance of at least one plasma hemostatic component, von Willebrand factor (VWF), that showed decreased sialylation [2]. Findings in mice lacking the ASGPR demonstrated that, during sepsis, the receptor removed components of hemostasis (VWF and platelets) that had been desialylated by bacterial neuraminidase and thereby allowed hemostatic adaptation that moderated disseminated intravascular coagulation and improved host survival [3]. The ASGPR may therefore be poised for rapid clearance of plasma glycoproteins that, for whatever reason, show decreased or abnormal sialylation.

The highest level of expression of the ASGPR is in the liver, in which it is located on the sinusoidal face of hepatocytes [4]. Low-level expression has been shown in various other cell types, such as, peritoneal macrophages in rat [5], human intestinal epithelial cells [6], mouse testis [7], and the rat thyroid gland [8]. The role of the receptor at these extrahepatic sites is uncertain. The galactosyl homeostasis theory proposes that the balance of both Gal and GalNAc glycoconjugates is important for normal tissue physiology [1, 9]; whether or not expression of the ASGPR at these other sites represents a need for localized constitutive control of such glycoconjugates is not known.

A major area of interest focussing on the ASGPR is the targeted gene transfer/delivery of drugs to the liver [10, 11]. For example, using GalNAc as a ligand on a siRNA-containing complex, the latter was successfully targeted to hepatocytes after simple intravenous injection into the tail vein in mice [11]. Moreover, siRNA-mediated knock-down of targeted genes was demonstrated within the hepatocytes. Besides its potential importance in allowing liver-specific drug targeting [12], the ASGPR is a key factor in the design and administration of glycoprotein pharmaceuticals more generally: the activity of the receptor can impact upon drug half life and thereby the window of therapeutic efficacy.

Human ASGPR is composed of two types of subunit: a major subunit (asialoglycoprotein receptor 1, ASGR1) and minor subunit (asialoglycoprotein receptor 2, ASGR2) [13]. Both subunits are type II, single pass proteins that broadly comprise a cytoplasmic domain, transmembrane domain, and extracellular carbohydrate recognition domain (CRD) [14, 15]. The subunits may exist as ASGR1-ASGR2 heterooligomers, ASGR1homotrimers and homotetramers, and ASGR2 homodimers and homotetramers. These different quaternary forms may allow for functional differences, such as, substrate specificity or rate of endocytosis [1, 16].

The genes encoding ASGR1 and ASGR2 (*ASGR1* and *ASGR2, *resp.) are located on the short arm of autosome 17, approximately 58.6 kilobases (kb) apart. The genes are evolutionarily related but differ significantly in their structural organization: *ASGR1* comprises 8 exons and is approximately 6 kb long, *ASGR2* contains 9 exons occupying 13.5 kb of DNA [14, 15].

Until recently, *ASGR1* was thought to yield one transcript encoding a single protein. However, in 2010, Liu et al. demonstrated the existence of two alternatively spliced transcripts. The longer transcript, T1, contains all 8 exons and is by far the more abundant; it encodes full-length ASGR1 (isoform a, 291 aminoacids). The shorter transcript, T2, has an in-frame deletion of exon 3 resulting in the loss of 39 residues (isoform b, 252 amino acids). Isoform b lacks the transmembrane domain and is secreted as a soluble protein [17].

*ASGR2* gives rise to five transcripts (TH2′, T1, T2, T3, and T4) encoding four isoforms (a to d) that contain different in-frame deletions arising from alternative exon splicing events [15]. Isoforms a and c contain 5 aminoacids that serve as a proteolysis cleavage signal near the junction between the transmembrane domain and the CRD [18]. Cleavage at this site results in secretion of the CRD as a soluble protein [18, 19]. Isoforms b and d lack this signal therefore they are not proteolytically cleaved but rather remain membrane bound where they may oligomerize with ASGR1 isoform a to form native ASGPR at the cell surface.

The secreted forms of ASGR1 and ASGR2 are able to associate into soluble ASGPR [17, 19]. There is evidence to suggest that the soluble receptor may bind free substrates in the circulation and carry them to the liver for uptake and degradation [17]. Aside from membrane attachment/secretion, it is not known whether further functional variation, such as, substrate specificity, may be associated with the different isoforms of ASGR1 and ASGR2.

Based on the observation above that the ASGPR is expressed in rat peritoneal macrophages, it was considered possible that monocytes, the lineage precursor of tissue macrophages, may also express the receptor. To the best of our knowledge, this has not been reported in human monocytes. The present study investigated the expression of *ASGR1* and *ASGR2* in human peripheral blood monocytes. The data showed that both *ASGR1* and *ASGR2* are expressed in human monocytes in the circulation, that expression cannot be detected in lymphocytes and granulocytes, that the transcripts of both genes differ between individuals and that, in a given individual, the transcription profile for *ASGR2* is restricted to one of two patterns. The findings are of potential importance for health and disease in a variety of disciplines.

## 2. Design and Methods

### 2.1. Nomenclature

Genes, transcripts, and proteins are referred to by their formal scientific names as listed in the National Centre for Biotechnology Information (NCBI) [20], which complies with standardized international nomenclature.  Table 1 provides a comparison with alternative names widely used in ASGPR literature. Gene sizes, exon numbering, and so forth were obtained from the reference sequences listed in Table 1.

### 2.2. Blood Samples

EDTA-anticoagulated whole blood in excess of that required for routine blood tests was anonymized and handled in accordance with Medical Research Council guidelines [21]. The study was reviewed by the local research ethics committee. All samples had normal haematological parameters.

### 2.3. Separation of White Blood Cells

#### 2.3.1. Stage 1

 Depletion of platelets. Citrate- or EDTA-anticoagulated whole blood (5 mL) was centrifuged at 200× g for 10 minutes, ambient temperature to prepare platelet-rich plasma (PRP). Two-thirds of the volume of the PRP was carefully removed from above the buffy coat, centrifuged at 1800× g for 10 minutes to pellet the platelets, and then the supernatant plasma returned to the original blood sample. This process was repeated three times.

#### 2.3.2. Stage 2

 Separation of cells. After the depletion of platelets as described above, the blood sample was loaded onto a discontinuous Histopaque (Sigma Aldrich, Dorset, UK) gradient comprising Histopaque 1119 (2 mL, lower layer) and Histopaque 1077 (3 mL, upper layer). Following centrifugation at 800× g for 1 h at ambient temperature, the cells at the interface between the plasma and Histopaque 1077 (monocytes and lymphocytes) were harvested; the cells at the interface between Histopaque 1077 and 1119 (principally granulocytes with a small proportion of lymphocytes) were also harvested.

Harvested cells were washed by resuspension in 10 mL phosphate buffered saline pH 7.2 (PBS) followed by centrifugation at 250× g for 10 minutes at ambient temperature. The cell pellet was resuspended in PBS (0.7 mL) and an aliquot (0.2 mL) was diluted 1 : 1 (v/v) with PBS and used for determination of cell counts on a Pentra 120 (Horiba ABX, Montpellier, France).

### 2.4. Cell Sorting Using Flow Cytometry

Citrate- or EDTA-anticoagulated whole blood (5 mL) was depleted of platelets as described above (Separation of White Blood Cells). The blood sample (3 mL) was then loaded onto Histopaque 1077 (3 mL) and centrifuged to separate the white blood cells (WBC) (400× g, 30 min, ambient temperature). The WBC interface was harvested, washed, and an aliquot used for determination of cell counts as described above (Separation of White Blood Cells). 

The remaining cells, suspended in PBS, were separated according to forward scatter and side scatter light characteristics using a MoFlo high speed cell sorter (Beckman Coulter, High Wycombe, Bucks, UK). Gating was restrictive rather than permissive: each gate was set only for the core population of cells that had the required scatter characteristics. In particular, the lymphocyte gate was set to minimise the possibility of small monocytes being harvested. Purity checks were subsequently performed by reanalysis of aliquots of the sorted cells in the flow cytometer; purity was typically around 98%.

### 2.5. Cell Culture

All reagents and consumables for cell culture were supplied by Invitrogen Life Technologies Ltd, Paisley, Ireland. HepG2 (human hepatocyte carcinoma) [22] and THP1 (human acute monocytic leukemia) [23] cells were cultured in RPMI1640 medium + L-Glutamine, supplemented with 10% (v/v) fetal calf serum and containing penicillin (50 units/mL) and streptomycin (50 *μ*g/mL). Cells were grown at 37°C in water-saturated air (95%, v/v), CO_2_ (5%, v/v) in 25 cm^2^ flasks. HepG2 cells (adherent cell line) were liberated from the flask wall mechanically using a plastic scraper and were separated by exposure to shear force. THP1 cells (nonadherent) were harvested directly from the culture medium. Cells were counted using FastRead 10 disposable hemocytometers (Immune Systems, UK). HepG2 has been shown by others to express high levels of the Ashwell receptor [24] and was used as a positive control in all relevant experiments.

### 2.6. RNA Extraction and Reverse Transcription

RNA was extracted using RNeasy kits (Qiagen, West Sussex, UK) according to the manufacturer's instructions. Yield was determined spectrophotometrically and then 1.0 *μ*g was reverse transcribed into cDNA using random hexamers and the High Capacity cDNA Reverse Transcription kit (Life Technologies Ltd/Applied Biosystems, Paisley, UK) according to the manufacturer's instructions.

### 2.7. Polymerase Chain Reaction

**Figure 1:**
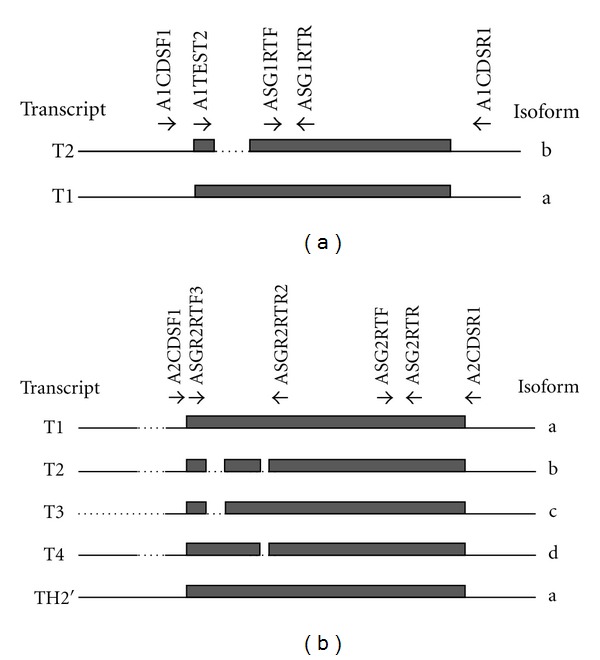
*ASGR1* and *ASGR2* transcripts and encoded protein isoforms. (a) *ASGR1*. (b) *ASGR2*. In both panels solid lines indicate nucleotide sequence that is present in a transcript, grey boxes represent coding sequence, dotted lines indicate sequence that is not present in a transcript. Arrows indicate location and direction of primers used in RT-PCR. Data for transcripts and isoforms are taken from NCBI [14, 15].

Standard polymerase chain reactions (PCRs) typically contained approximately 30 ng cDNA in a mixture of dNTPs (100 *μ*mol/L each), Tris-HCl pH8.0 (10 mmol/L), MgCl_2_ (1.5 mmol/L), *Taq* DNA polymerase (1U) (Applied Biosystems, Warwickshire, UK), and primers (Figure 1) in a final volume of 25 *μ*L. All primers were used at 0.5 *μ*mol/L. PCR was done using a 2720 DNA Thermal Cycler (Applied Biosystems, Warwickshire, UK).

The primer sequences (5′ to 3′) were as follows:

 ASG1RTF gaaagatgaagtcgctagagt
 ASG1RTR aggctccgcaggtcagacac
 A1CDSF1 gtagcgcgacggccagtactgaagaacctgggaatcagac
 A1CDSR1 cagggcgcagcgatgacagctcctcaccttcggaacatca
 A1TEST2 gaccaaggagtatcaagacctt
 ASG2RTF cacacctggtggtcatcaac
 ASG2RTR aattatctggctgagtgacag
 ASGR2RTF3 agctgagctcggaggaaaatg
 ASGR2RTR2 gcagctcggcttgcagctgtg
 A2CDSF1 gtagcgcgacggccagtcccagccctcagagcaacctca
 A2CDSR1 cagggcgcagcgatgactcaacagagaagccagagctggg
 B2MRTF tccgtggccttagctgtgct
 B2MRTR ccagtccttgctgaaagaca
 N13F  gtagcgcgacggccagt
 N13R cagggcgcagcgatgac

Primers were designed for RT-PCR according to the following criteria: (1) they flanked at least one intron; (2) they were specific for *ASGR1* or *ASGR2* transcripts, cross hybridization was not possible despite the sequence homology between the coding sequences of the two genes. N13F and N13R are, respectively, modified M13 universal and reverse sequencing primers as previously described [25]. Underlined nucleotides are not part of *ASGR1* or *ASGR2* sequence but are tails corresponding to N13F or N13R to facilitate sequence analysis.

For nested PCR, first-round synthesis was as above. The first-round product was then diluted 10-fold with water and 1 *μ*L of diluent was used as the substrate in the second round of PCR.

PCR conditions were as follows:

  Standard PCR: 94°C for 30 s; 60°C for 30 s; 72°C for 1 min.
 Nested PCR: 1st round 94°C for 30 s; 60°C for 30 s; 72°C for 1 min 30 s.
 Nested PCR: 2nd round 94°C for 30 s; 60°C for 30 s; 72°C for 1 min.

Routinely, 30 cycles of amplification were used; however, for real time PCR, 55 cycles were employed.

### 2.8. Real-Time Quantitative PCR

*ASGR1* and *ASGR2* transcript levels were measured using LightCycler FastStart DNA Master SYBR Green I (Roche Products Ltd, Hertfordshire, UK) according to the manufacturer's instructions, with 3 mmol/L MgCl_2_ final concentration in the reaction. Reactions contained 2 ng cDNA per 20 *μ*L and relevant primers (Figure 1). Primers were used at 0.5 *μ*mol/L. Analyses were done in duplicate in a LightCycler II using LightCycler v4.0 software (Perkin Elmer-Applied Biosystems, Warwickshire, UK) using the following conditions: 94°C for 10 min followed by 55 cycles comprising 94°C for 10 s; 64°C for 5 s; 72°C for 10 s.

### 2.9. Nucleotide Sequence Analysis

PCR products were purified using the HighPure kit (Boehringer Mannheim, Nottingham, UK) and then sequenced using BigDye3.1 according the manufacturer's instructions (Perkin Elmer-Applied Biosystems, Warrington, UK). Sequencing primers were either the 5′ PCR primer, 3′ PCR primer, N13F or N13R according to the amplification product. Sequencing reaction products were purified using QIAquick PCR purification columns (Qiagen, West Sussex, UK) and then electrophoresed on an Applied Biosystems 3130XL Genetic analyser.

### 2.10. Gel Electrophoresis of PCR Products

Agarose gel electrophoresis: the analyses used 2% (w/v) gels and 1x TBE buffer (tris (90 mmol/L), boric acid (90 mmol/L), EDTA (1.25 mmol/L), pH 8.0); visualization was done using ethidium bromide staining and UV light.

Polyacrylamide gel electrophoresis: gels comprised polyacrylamide (total acrylamide = 5%, w/v; cross link = 3.3%, w/v) and 1x TBE buffer, visualization was done using silver staining [26].

The DNA size standard pBR322/*Msp*I (New England Biolabs, Hertfordshire, UK) was used for all electrophoretic analyses.

## 3. Results

### 3.1. *ASGR1* and *ASGR2* Are Expressed in Peripheral Blood Monocytes

**Figure 2:**
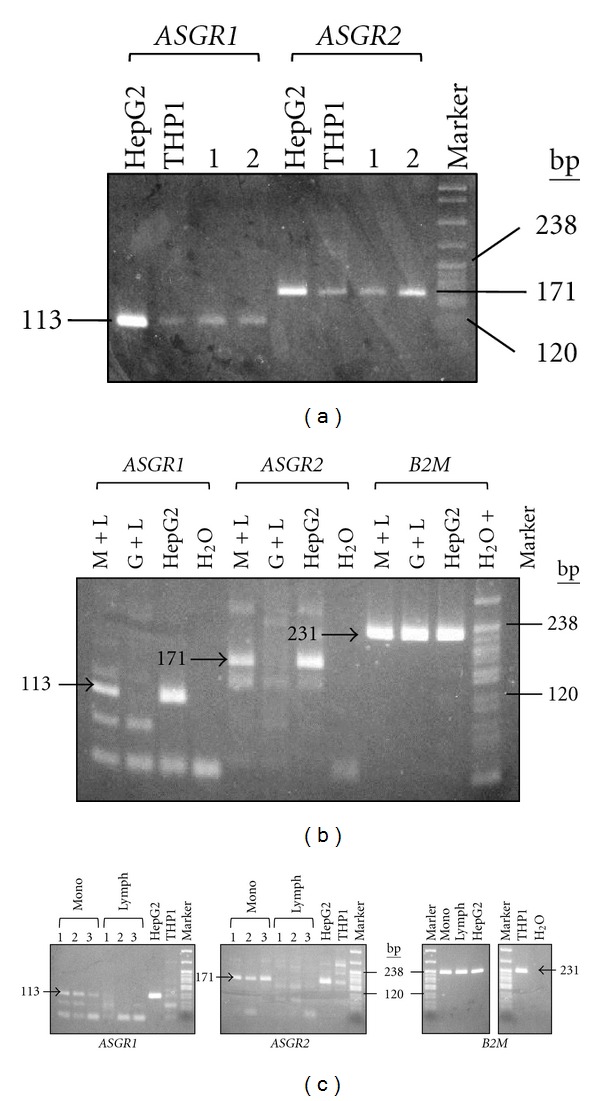
Detection of *ASGR1* and *ASGR2* transcripts in peripheral blood mononuclear cells and localization to monocytes. Transcripts were detected using RT-PCR, the presence of *ASGR1* transcripts was indicated by a 113 bp amplification product (primers ASG1RTF + ASG1RTR) and *ASGR2* transcripts by a 171 bp product (primers ASG2RTF + ASG2RTR). HepG2 was used as a positive control and water as a negative control. Where necessary, *β*-2-microglobulin (*B2M*) was used as a reference to demonstrate equivalent amplification for each RNA preparation, (primers B2MRTF + B2MRTR, product 231 bp). (a) Analysis of RNA from monocyte cell line THP1 and PBMCs from two unrelated individuals (1 and 2). *ASGR1* and *ASGR2* transcripts were detected in all cases. (b) Analysis of RNA from peripheral blood cell fractions: monocytes + lymphocytes (M + L) and granulocytes + lymphocytes (G + L). *ASGR1* and *ASGR2* transcripts were detected in M + L but not in G + L. (c) Analysis of RNA from cell-sorted monocytes (mono, lanes 1–3 triplicate analyses) and lymphocytes (lymph, lanes 1–3 triplicate analyses) (data for water control not shown for *ASGR1* and *ASGR2*). The data were obtained from a single experiment involving several gels: these are indicated by separate gel windows. For each panel, analysis was done using agarose gel electrophoresis with pBR322/*Msp*I size standard (“Marker”, 238 bp and 120 bp indicated).

Initially, peripheral blood mononuclear cells (PBMCs) were harvested and screened for *ASGR1* and *ASGR2* expression without fractionation into monocytes/lymphocytes/granulocytes. RT-PCR of RNA extracted from PBMCs demonstrated the presence of both *ASGR1* and *ASGR2* transcripts (Figure 2(a)). These were also detected in the monocyte cell line THP1 (Figure 2(a)).

Following these initial findings, PBMCs were fractionated in order to localize the cells in which *ASGR1* and *ASGR2* expression was present. Histopaque gradients allowed separation of cells into monocytes + lymphocytes (M + L) and granulocytes + lymphocytes (G + L). These cell preparations typically contained 10% monocytes/90% lymphocytes (M + L) and 94% granulocytes/10% lymphocytes (G + L) (data not shown).

Using RT-PCR, *ASGR1* and *ASGR2* transcripts were detected in RNA from the M + L fraction but not in RNA from the G + L fraction (Figure 2(b)). These findings were reproducible for blood samples taken from 3 different individuals (data not shown). The G + L fraction did not yield a product for the transcripts even after 50 cycles of RT-PCR amplification (Figure 2(b)). Some nonspecific products were obtained as a result of this high number of cycles; however, these did not interfere with the main result of the experiment (Figure 2(b)).

Because lymphocytes were common to both cell fractions, and because the G + L fraction was reproducibly negative for *ASGR1* and *ASGR2* transcripts, the data suggested that expression of the two genes was localized to monocytes. To confirm this, monocytes and lymphocytes were formally cell sorted using flow cytometry, RNA extracted and screened using RT-PCR. Monocytes gave a positive result for both *ASGR1* and *ASGR2* transcripts, whilst lymphocytes did not give a PCR product, even after 50 cycles of amplification (Figure 2(c)). This high cycle number resulted in the burst-through of some nonspecific products; however, these did not interfere with the interpretation (Figure 2(c)). These data provide strong evidence for expression of *ASGR1* and *ASGR2* in monocytes but not in lymphocytes or granulocytes.

### 3.2. Monocyte *ASGR1* Transcripts Differ between Individuals

**Figure 3:**
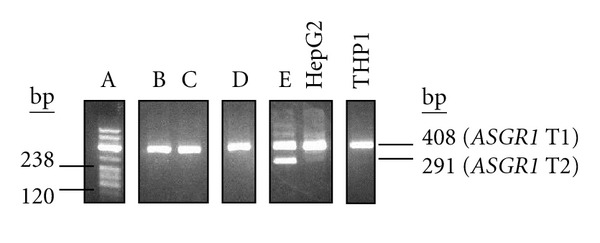
*ASGR1* transcript profiles in different individuals. Nested PCR was used to detect *ASGR1* transcripts T1 and T2 (first round primers A1CDSF1 + A1CDSR1; second round A1TEST2 + ASG1RTR). A product of 408 bp represented T1, present in RNA from the M + L cell fraction of five individuals (A–E) and also in HepG2 and THP1; a 291 bp product indicated the presence of T2 (individual E, and, very faintly, in HepG2). The RT-PCR product for individual A was coloaded with the DNA size standard (pBR322/*Msp*I, 238 bp and 120 bp sizes indicated). Analysis was done using agarose gel electrophoresis. The figure is a composite of various data (indicated by individual gel windows).

Two transcripts have been described for *ASGR1* [17]. The longer transcript (T1) encodes isoform a (full-length ASGR1), the shorter transcript (T2) has an in-frame deletion of 117 nucleotides in the coding sequence resulting in a shorter isoform (isoform b) (Figure 1(a)). The latter lacks the transmembrane domain and is secreted as a soluble protein. In the liver, T1 has been shown to be the predominant isoform, with very little T2 detectable [17]. Using a nested PCR approach to screen RNA from the M + L cell fraction of peripheral blood, different individuals showed the presence of either T1 or of both T1 and T2 (Figure 3). Among the five individuals screened, none showed the presence of transcript T2 on its own. In combination with the data in Figure 2 (which showed that *ASGR1* transcripts are detected in monocytes and not lymphocytes), these data suggest that the monocyte transcription profile for *ASGR1* differs between individuals. THP1 gave a result consistent with the presence of T1 (Figure 3).

### 3.3. Monocyte *ASGR2* Transcripts Differ between Individuals

**Figure 4:**
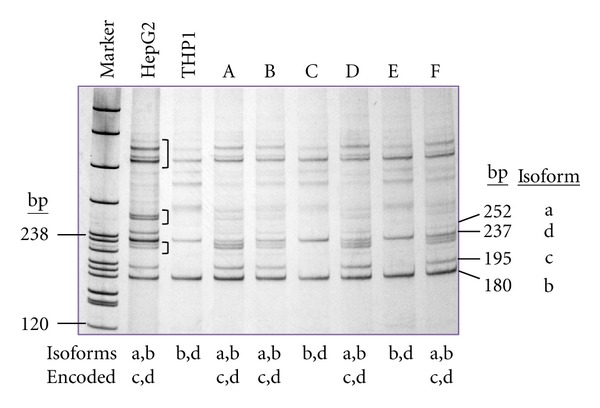
*ASGR2* transcript profiles in different individuals. Primer pair ASGR2RTF3 + ASGR2RTR2 gave RT-PCR products of different length (arrowed) according to the *ASGR2* transcripts present. RNA extracted from the M + L cell fraction of 6 different individuals (A–F) gave either a profile consistent with transcripts encoding all isoforms (a, b, c, and d) or just with isoforms b and d. The liver cell line HepG2 showed all transcripts, the monocyte cell line THP1 showed only transcripts encoding isoforms b and d. The analysis was done using polyacrylamide gel electrophoresis. DNA size reference was pBR322/*Msp*I (“Marker”, 238 bp and 120 bp sizes indicated). In addition to the expected products, additional major bands were visualized on polyacrylamide gel electrophoresis of the RT-PCR screen for *ASGR2* transcripts (bracketed bands). It was considered possible that these represented heteroduplexes arising by cross-hybridization of complementary strands of the closely homologous true PCR products. To test this, each candidate heteroduplex band was excised from the gel, eluted into water, reamplified using the primers used in the initial PCR and the products electrophoresed on polyacrylamide. The results confirmed that the additional bands were hybrid duplexes containing complementary strands from nonidentical true PCR products: each hybrid yielded products consistent with amplification of two different template strands (data not shown).

Five transcripts have been described for *ASGR2*, giving rise to four protein isoforms (Figure 1(b)) [15]. Using a RT-PCR designed to detect transcripts encoding different isoforms, the product profiles obtained for RNA extracted from the Histopaque M+L cell fraction differed among individuals. Two distinct profiles were obtained, the first corresponded to transcripts encoding isoforms a, b, c, and d, the second to transcripts encoding isoforms b and d only (Figure 4). ASGR2 isoforms a and c can give rise to soluble protein via proteolysis between the transmembrane domain and the CRD [18]. Isoforms b and d lack the proteolysis site and cannot produce the soluble form. The data therefore indicate that in some individuals, monocytes may produce both soluble and membrane-bound ASGR2, whilst in others, the soluble form is not produced by these cells.

### 3.4. Real-Time Quantitative PCR

**Figure 5:**
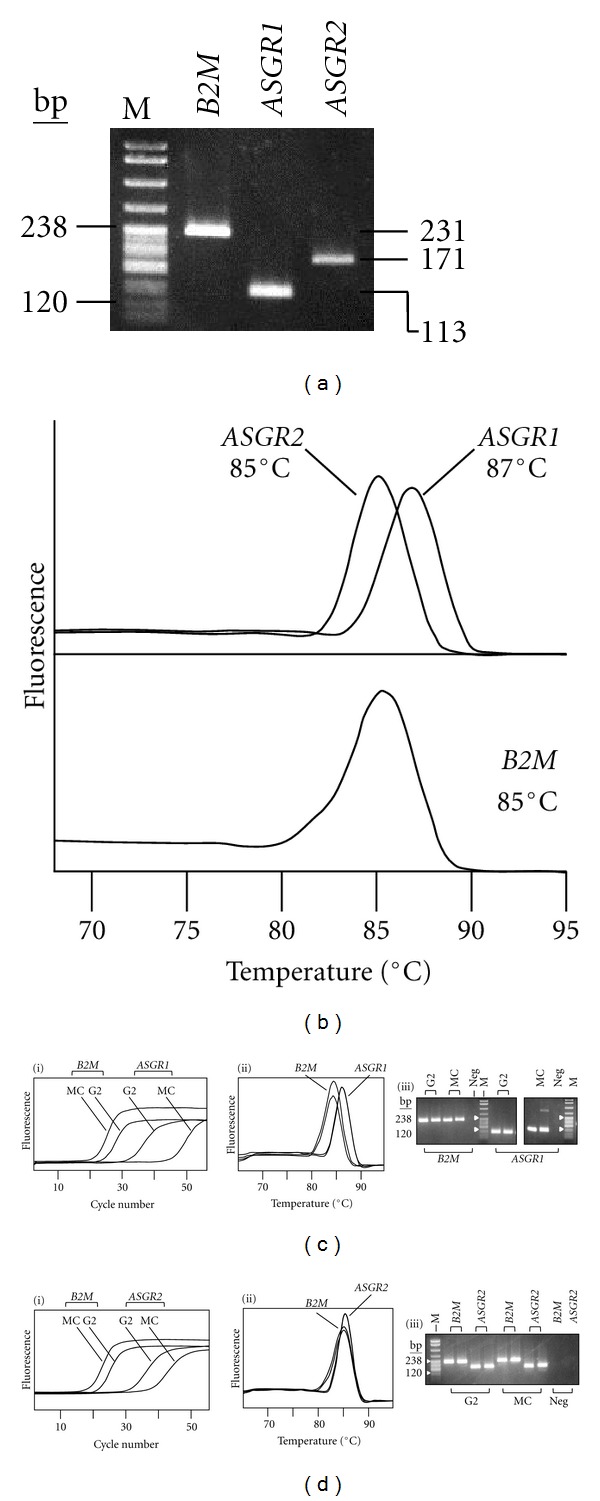
Real-time PCR analyses. (a) Agarose gel electrophoresis of products from optimized real-time PCRs for *ASGR1* (113 bp, primers ASG1RTF + ASG1RTR), *ASGR2* (171 bp, primers ASG2RTF + ASG2RTR), and *B2M* (231 bp, primers B2MRTF + B2MRTR). Substrate was HepG2 cDNA. (b) Melting curve analysis of real-time PCR products from panel (a). The Tm for each product is indicated. (c and d) Relative quantification of *ASGR1* (c) and *ASGR2* (d) transcripts in cell-sorted monocytes (MC) compared with HepG2 (G2). *B2M* was used as the reference. (i) Fluorescence profiles obtained during real-time PCR. Samples were analyzed in duplicate, for clarity only one of each duplicate is shown. (ii) Melting curve analysis for the real-time PCR products in (i). (iii) Agarose gel electrophoresis of real-time PCR products (duplicate analyses shown). Neg indicates real-time PCR control containing water instead of nucleic acid template. In panels a, c(iii), and d(iii), M denotes pBR322/*Msp*I size standard (238 bp and 120 bp sizes indicated).

Real-time PCR was optimized for primer pairs ASG1RTF + ASG1RTR, ASG2RTF + ASG2RTR (which, respectively, amplify all *ASGR1* and all *ASGR2* transcripts) and B2MRTF + B2MRTR (which amplify the reference target gene, *B2M*). For each primer pair, a specific product was obtained that had a characteristic melting curve (Figure 5).

Following PCR optimization, transcripts were measured using relative quantification, in which the target was *ASGR1* or *ASGR2*, the reference was *B2M*, the calibrator was HepG2, and the unknown was flow sorted monocytes or THP1. *B2M* is a recommended reference target for quantitative PCR, having the advantages of a single transcript, no processed pseudogene and expression at similar levels in a wide range of tissues [27]. Flow-sorted monocytes and THP1 cells gave similar results: relative to expression in HepG2, *ASGR1* transcripts were between 1.53*E*-07 to 7.53*E*-06-fold less, whilst *ASGR2* transcripts were between 7.16*E*-05 and 3.78*E*-04-fold less. Figure 5 illustrates relative quantification of *ASGR1* and *ASGR2* transcripts in cell-sorted monocytes. The ratio of expression of *ASGR1* : *ASGR2* in monocytes and the monocyte cell line THP1 was approximately 1 : 100.

### 3.5. Nucleotide Sequence Analysis

To ascertain whether THP1 would be a suitable cell line for future ASGPR studies in monocytes, the coding sequences of the *ASGR1* and *ASGR2* transcripts produced by these cells were determined. A nested PCR strategy was used as follows: the first round of synthesis amplified the entire coding sequence plus some 5′ and 3′ flanking sequence and then second round PCRs amplified nested portions within the first round product. The nested portions were sequenced in both directions. For *ASGR2*, one of the nested portions contained the region that differed between transcripts and gave products of different size according to the transcript. These products were electrophoresed, excised from the gel, reamplified individually, and then sequenced, to give the sequence of each of the coding regions that differed between transcripts.

THP1 *ASGR1* and *ASGR2* transcripts corresponded with previously described splice variants found in the liver (data not shown). The findings demonstrate that THP1 cells have the capacity to transcribe and process correctly, transcripts encoding functional isoforms of the ASGPR. Taken together with the results in Figures 3 and 4, the data provide evidence that monocytes express correctly processed transcripts from both *ASGR1* and *ASGR2* and thereby have the potential to produce functional ASGPR.

The coding sequence for *ASGR1* in THP1 differed from the NCBI reference sequence (Table 1) by a single nucleotide: c.267G>A, for which the monocyte cell line was homozygous (data not shown). This change is silent at the protein level (codon 89, AAG, changes to AAA, both encoding lysine). The change is a naturally occurring variant (rs55714927) listed in the international SNP database (dbSNP) [28]. The THP1 *ASGR2* coding sequence for any transcript did not differ to the corresponding NCBI reference sequence (Table 1) (data not shown).

## 4. Discussion

In this study, expression of *ASGR1* and *ASGR2* was demonstrated in peripheral blood monocytes. For both genes, transcript profiles were obtained that corresponded with known splice variants found in the liver. To the best of our knowledge, this is the first report of the expression of correctly processed transcripts of *ASGR1* and *ASGR2* by human monocytes. In rat, a transcript encoding an asialoglycoprotein-binding protein was isolated from a peritoneal macrophage cDNA library and was highly homologous to that of the rodent hepatic ASGPR [5]. The data were interpreted to indicate that the rodent macrophage asialoglycoprotein-binding protein and the liver ASGPR were encoded by related genes [5]; however, it now seems possible that alternative splicing may underlie the differences. The findings of the present study do not suggest expression of alternative ASGPR-related genes in monocytes, rather they are entirely consistent with transcription of *ASGR1* and *ASGR2*, as occurs in the liver.

The various transcripts of *ASGR1* and *ASGR2* encode different isoforms of the proteins (Figure 1). It is not known whether the isoforms differ in their specificity or functionality, however it is predicted that certain isoforms have the potential to give rise to soluble forms of each protein. *ASGR1* transcript T2 has an in-frame deletion of 117 bp that encode the transmembrane domain; the resulting isoform “b” therefore is soluble and secreted [17]. *ASGR2* transcripts T1, TH2′ and T3 encode isoforms “a” (T1 and TH2') and “c” (T3) which contain a proteolysis signal that, upon cleavage, gives rise to soluble protein [18]. Transcripts T2 and T4, respectively, encode isoforms b and d which lack the proteolysis signal and are membrane bound. The data presented here indicate that monocytes are able to express transcripts encoding soluble and membrane-bound isoforms of the ASGPR proteins. *ASGR2* was found to give rise to two transcript profiles, only one of which was present in the monocytes from any given individual. One profile corresponded with transcripts encoding both soluble and membrane-bound ASGR2 isoforms (a, b, c, and d), the other with membrane-bound ASGR2 isoforms (b and d) only. This novel finding indicates a fundamental difference between individuals. The underlying basis for this and whether or not it has physiologic significance in health or disease merit further exploration. 

The level of *ASGR1* and *ASGR2* expression was considerably less in monocytes and the monocyte cell line THP1 compared with the liver cell line HepG2. It should be borne in mind that HepG2 expresses high levels of the receptor [24] and the monocyte expression was relative to this. That monocyte transcripts were not rare within the cells was indicated by the fact that they were readily detected using RT-PCR at routine cycle numbers. Low level of expression of the ASGPR has been reported previously in certain nonhepatic tissues (rat macrophages [5], human intestinal epithelial cells [6], mouse testis [7], and rat thyroid gland [8]). It would be relevant to explore whether *ASGR1* and *ASGR2* transcription alters upon monocyte activation.

The relative expression of *ASGR1* and *ASGR2* in monocytes and THP1 (1 : 100) differed notably from ratios reported in liver (1 : 2 [1] and 1 : 6 [16]). The difference may be real, or it may reflect differences in methodology between studies or possibly a difference in the amplification efficiency of the primer pair used for *ASGR1* compared with that used for *ASGR2*. The true relative expression of the two genes by monocytes therefore remains to be established.

An important question arising from these results is whether monocytes translate *ASGR1* and *ASGR2* transcripts and produce functional ASGPR. The restricted expression of the genes in monocytes, but not in lymphocytes or granulocytes, suggests the transcripts are not produced randomly but specifically. Based on the fundamental principles of biology, this is likely to be for translation, it is difficult to think why else different transcripts encoding different isoforms may be produced by one specific cell type in the blood. The restricted tissue specificity for ASGPR expression in the body, and the lower expression level observed at the nonhepatic locations, could signal a specific role or function at those sites. In the case of monocytes, the receptor could, at the very least, serve a scavenger function where there is infection or tissue injury.

Expression of correctly processed transcripts of *ASGR1* and *ASGR2* by circulating monocytes has potentially significant implications in several important areas. These can be grouped into two main themes: normal physiology and drug design. If monocytes express functional ASGPR, they may contribute towards the normal physiological processes undertaken by the hepatocyte receptor. Whilst hepatocyte ASGPR is localized to the liver and relies upon the circulation to deliver ligands to it, the monocyte receptor would represent a mobile pool that can reach ligands in most parts of the body. Monocyte ASGPR may additionally interact with ligands in the blood, as does liver ASGPR. Thus, the monocyte receptor, if it is produced, may overlap functionally with that of the liver but may have additional physiological roles elsewhere in the body.

Hepatic ASGPR has been shown to be directly involved in the normal turnover of an important protein of primary hemostasis, VWF. Studies in knock-out mice indicated that the ASGPR is engaged in the constitutive control of VWF level and can, additionally, remove hemostatic components (VWF and platelets) desialylated by bacterial neuraminidase, a possible survival mechanism in disseminated intravascular coagulation [3]. The results in these studies were ascribed to the activity of the liver receptor; however, our data raise the possibility that monocytes may contribute to the relevant physiological processes via the ASGPR.

The findings have implications for the design of drugs targeting the liver via the ASGPR and drugs that are cleared by this receptor [29, 30]. Various strategies have been used, for example, conjugation of drug with galactosyl terminating molecules, as has been done with antiviral nucleoside analogues in the treatment of chronic viral hepatitis [31]. Besides drug targeting, strategies to deliver genes or other nucleic acids to the liver have also made use of the ASGPR [10–12]. Relatively recently, successful *in vivo *delivery of siRNAs to the liver in mice has been achieved via simple intravenous injection employing ASGPR-specific conjugates containing GalNAc [11]. Pharmacological targeting of the ASGPR could not be considered to be predominantly liver-specific if monocytes express the receptor.

## 5. Conclusions

*ASGR1* and *ASGR2* are expressed in peripheral blood monocytes in humans. For both genes, monocytes have the ability to produce correctly spliced transcripts encoding each of the known protein isoforms. However, there are interindividual differences in the transcript profiles; of particular note, despite several possible *ASGR2* transcripts, just two combinations were found to occur. This observation indicates that, in any given individual, *ASGR2 *mRNA splicing is restricted to one of two profiles. Quantitative studies indicate that expression of both *ASGR1* and *ASGR2* is lower in monocytes than in the liver, but none-the-less is readily detected in monocytes (in comparison to other blood cells in which expression could not be demonstrated). The liver may represent a static site to which blood carries glycoconjugates for ASGPR-mediated removal, whilst monocytes may represent a mobile pool that can reach sites where the activity of the receptor is needed.

The data presented here open various important avenues for future research, a notable one of which is the measurement and characterization of monocyte ASGPR protein. The results for the cell line THP1 indicated it could be used as a model system for the investigation of monocyte ASPGR function and activity. Monocytes play a pivotal role in inflammation and immunity, the finding of ASGPR transcripts within these cells offers new insight into their biochemistry and functional potential.

## Figures and Tables

**Table 1:** ASGR1 and ASGR2 nomenclature and reference sequences.

Gene and transcript (NCBI)	Protein and isoform (NCBI)	Protein and isoform alternative name	Gene and transcript reference sequences (NCBI)	Protein and isoform reference sequences (NCBI)
*ASGR1*	ASGR1	H1	AC_000060.1	not applicable

T1	a	H1a	NM_001671.4	NP_001662.1
T2	b	H1b	NM_001197216.2	NP_001184145.1

*ASGR2*	ASGR2	H2		

T1	a	H2a	NG_029064.1	not applicable
T2	b	H2c (L-H2)	NM_001181.4	NP_001172.1
T3	c	None	NM_080913.3	NP_550435.1
T4	d	H2b	NM_080914.2	NP_550436.1
TH2^′^	a	H2a	NM_001201352.1NM_080912.3	NP_001188281.1NP_001172.1

NCBI abbreviates National Centre for Biotechnology Information [20].
